# Typeface Reveals Spatial Economical Patterns

**DOI:** 10.1038/s41598-019-52423-y

**Published:** 2019-11-04

**Authors:** Ruixian Ma, Wei Wang, Fan Zhang, Kyuha Shim, Carlo Ratti

**Affiliations:** 1 Senseable City Laboratory, Massachusetts Institute of Technology, Cambridge, MA 02139 USA; 2 Alibaba Group, Hangzhou, 310000 China; 3 School of Design, Carnegie Mellon University, Pittsburgh, PA 15213 USA

**Keywords:** Environmental social sciences, Socioeconomic scenarios

## Abstract

Understanding the socioeconomic and demographic characteristics of an urban region is vital for policy-making, urban management, and urban planning. Auditing socioeconomic and demographic patterns traditionally entails producing a large portion of data by human-participant surveys, which are usually costly and time consuming. Even with newly developed computational methods, amenity characteristics such as typeface, color, and graphic element choices are still missing at the city scale. However, they have a huge impact on personalized preferences. Currently, researchers tend to use large-scale street view imagery to uncover physical and socioeconomic patterns. In this research, we first propose a framework that uses deep convolutional neural network to recognize the typeface from street view imagery in London. Second, we analyze the relationship between 11 typefaces and the average household income in 77 wards of London. The result show that the typefaces used in the neighborhood are highly correlated with economic and demographic factors. Typeface could be an alternative metric to evaluate economic and demographic status in large-scale urban regions. More generally, typeface can also act as a key visual characteristic of a city.

## Introduction

Researchers and policy-makers have been studying socioeconomic patterns for decades. Uncovering underlying socioeconomic patterns such as household income distribution could facilitate the effective allocation of city resources, further fulfilling the needs of urban dwellers.

The income distribution is shown to be related to where residents with different income levels live^1^. Three factors are generally used to measure the living preferences, namely, accessibility, space, and environmental amenities^2^. Accessibility is the distance to shops or companies’ locations. Pioneers, such as William Alonso^3^, measure the accessibility based on t/q. Later, Brueckner *et al*. observed that people tend to move to neighborhoods where amenities fit their expenditure requirements^4^. Environmental amenities include natural features, neighborhood characteristics, and so on. To date, the computational method using publicly available data to extract natural features and predict socioeconomic data is causing phenomenon impact. Jean *et al*. accurately predicted spatial poverty results between 2013 to 2015 through five African countries using satellite imagery (Google Static Maps)^5^. Apart from the natural features, Gebru *et al*. used vehicles extracted from Google Street View images to predict income, race, education, and voting patterns. The study successfully validated that socioeconomic patterns can be predicted by objective characteristics from neighborhoods^6^. Similarly, by extracting scene information only from street view images, a deep learning model can predict daily human mobility patterns at urban streets^7^.

However, few studies have looked into individual characteristics within neighborhood characteristics, such as amenity characteristics. We assume that amenity characteristics matter for household income as well as other aspects. For example, London Covent Garden and Old Street have different neighborhood styles and amenity characteristics. People with similar income may prefer living in different areas because of different area characteristics. In other words, the quantity or accessibility of amenities cannot adequately represent incomes in different areas. Under certain circumstances, the amenity characteristic could better describe household incomes.

To the best of the authors’ knowledge, a study is still to be conducted to discover the patterns between amenity characteristics and household incomes at the city scale. It has long been accepted that typeface is one of the key elements of amenity characteristic^8^, and typeface that appears on signages and posters can also indicate people’s aesthetic preferences. Here, we propose the use of typeface to predict household income by only using publicly available data. Collecting city-scaled typeface usage still retains many challenges because identifying the thousands of typeface styles requires professional training. Therefore, the traditional methods of collecting demographic data, such as crowdsourcing or door-to-door study, will not apply to typeface data retrieval.

Computational text and typeface recognition methods have been proposed in recent years. Jaderberg *et al*. spotted text in natural image^9^. Wang and Chen recognized typefaces from wild scene backgrounds^10,11^. However, identifying typeface style from Google Street View images is more challenging because they have lower resolution and higher distortion than other sources of images, such as Flickr and Panoramio^12,13^. To address the issues discussed above, we propose a framework in this study. First, we generate training data in training the deep learning model to recognize typefaces by using Convolutional Neural Network(CNN)^14^. Second, we map the city-scaled typeface data to the corresponding geospatial properties for correlation analysis between typefaces and socioeconomics.

In this work, we collect 59,515 typefaces images from 748,471 Google Street View images in 77 wards in London. We take the distribution of typefaces and amenity types as the predictors and the household income (https://data.london.gov.uk/) as the response in a multivariate regression, obtaining a *R*^2^ of 0.552, which considerably outperformed the result only using the distribution of amenity types as the predictors (*R*^2^ = 0.297). Through a correlation analysis, we find that the typefaces are highly correlated with amenity categories. The result verifies some knowledge that could only be previously explained by a designer’s instinct. For example, designers tend to use Sans-Serif and Serif typefaces in finance-related industries. Our result shows that these two typefaces are the first two correlation coefficients related to finance. Furthermore, we use Spearman correlation to map 11 typefaces and household incomes. We find that the relationships between them vary a lot. For example, Serif is positively correlated with income, whereas Sans-Serif has a coefficient negatively correlated with income. Thus, different typeface selections could attract different household income residents. In summary, this research contributes to the following directions:

- Typeface types and most common amenity categories are correlated in a neighborhood scale.
- Typeface can act as a metric to measure socio-economic characteristics.

## Results

### Typeface impression

We collected 900 typefaces that frequently appear in urban streets, including Helvetica, Gill Sans, Times New Roman, etc. To obtain a better recognition accuracy and demonstrate a clearer relationship between typefaces and economical factors, we labeled 900 typefaces into the 11 most commonly used typeface classes^15,16^, such as Serif, Sans Serif, and Script. Considering that the weight of the font could change its visual influence^17^, we built two categories for each of Serif, Sans Serif, and Script, in terms of Regular and Bold. Owing to the fewer number of typefaces and their inconspicuous weight, we set Decorative, Casual, and Blackletter to only have a regular weight. Despite being a type of Serif typeface, Slab also has a squared stroke at the end; therefore, we set Slab as an individual class in our classification system.

We recognized 59,515 text images and their corresponding typefaces from 748,471 Google Street View images in central London. Sans-Serif is the most-used typeface in the city, which occupies over 25% the typeface database. This pattern makes sense to designers because Sans Serif is generally more friendly and preferred than the other typefaces^18,19^. In particular, both Helvetica and Gill Sans (Sans Serif category) frequently appear in London. Decorative and Serif are the second- and third-most popular typefaces, respectively.

**Figure 1:**
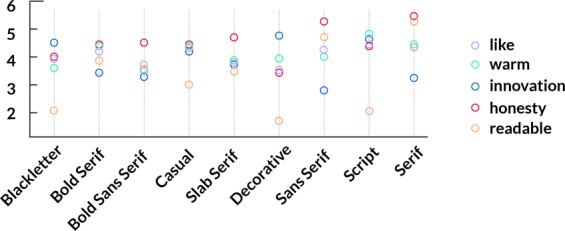
Nine-class typeface impressions.

Furthermore, as a typeface affects our perceptions, we followed Henderson’s^20^ typeface emotion experiment, which discovers potential trade-off impressions caused by typefaces. Then, we clustered them into our 9 out of 11 commonly used typeface classes. Some of the typefaces were already in our 900 typeface dataset. To demonstrate, Gill Sans, Arial, and Garamond are in Sans Serif. We asked five designers to map the rest of the typefaces scored by Henderson’s experiment. From Fig. 1, we can see that human perceptions of different typefaces vary drastically. For example, Serif had the highest score regarding honesty. This finding explains why Serif is extensively used for finance amenities. Sans-Serif has the lowest score regarding innovation, whereas Decorative has the highest innovation score because it always delivers an innovative and engaging feeling. In this connection, we believe that the presence and frequency of different typefaces is associated with local amenities and socio-economics.

### Correlation between typefaces and amenity types

How to choose the appropriate typeface in enhancing consumer purchasing behavior was examined by previous works, such as Doyle’s appropriate font choice study^21^ and Ulrich’s package design guidelines^22^. However, few studies discovered the relationships between amenity types and typefaces under spatial scenario. Consequently, we calculate the Spearman’s Rank correlation coefficient to quantify the relationship between typefaces and amenity data. In this case, we only use the sample pairs of a typeface and amenity type that matched with each other. In total, there are 5,238 amenities with their corresponding typefaces being obtained. Details about the matching approach is elaborated in method section.

**Figure 2:**
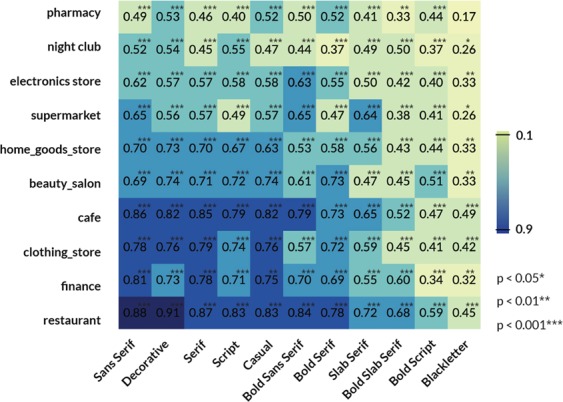
Correlation coefficients between typefaces and amenity categories. The x-, y-axis refer to the typeface types and amenities categories, respectively. The value represents the corresponding correlation coefficient between a pair of typeface and amenity category.

As demonstrated in Fig. 2, Sans Serif, Serif, and Decorative present high correlations with most of the amenity types. Indeed, our data also show that these three typefaces are the top three commonly used typefaces in the city. By comparing the correlation coefficients of different typefaces with the same amenity type, we can determine which typefaces are more preferred for this amenity type. For example, finance has a higher correlation with Sans Serif and Serif than other typefaces with the correlation coefficient of *ρ* = 0.81 and *ρ* = 0.78, respectively. This result indicates that finance-related industries, such as banks, prefer to use Serif and Sans Serif typefaces. For instance, the signage of Santander and HSBC uses Serif, and Bank of America uses Sans Serif. Through the overall observation of Figs. 1 and 2, we can better explain on the reasons why an amenity chooses such typefaces. It can also be interpreted that banks consider honesty and credible as an important impression. Notably, both Sans Serif and Serif are considerably correlated to finance because they have a high degree of honesty impression.

Another interesting example that can be discovered in Fig. 2 is that Serif has a high correlation coefficient with most amenity types. However, nightclubs are relatively less relevant with Serif typeface than most amenity types. This phenomenon happens presumably because the Serif typeface gives readable and honesty impressions, and these features are not what nightclubs typically attempt to deliver to their customers. By contrast, the top two correlated typeface types used in nightclubs are Script and Decorative, which all have higher innovation and warm impressions than the other typeface types.

The preliminary analysis and results demonstrated the potential usage of typefaces on the evaluation of local urban functions and socio-economics. In order to further examine the additional contribution of typefaces when the amenity information being controlled, we conducted multivariate regressions in the following part.

### How presence of typefaces associated with socio-economic characteristics

We adopted multivariate regression to explore how well typefaces can explain the variation of household incomes. As a control group, we also take amenity information in the regression analysis. In detail, we built three models with different variables to model the variation of household income. As is shown in Eqs. 1, 2 and 3.

By only taking amenity information as the explanatory variables, the household income is modeled as follows:

1 $$Amenity\,model(Model\,\mathrm{1):}\,log(Income)={\beta }_{0}+{\beta }_{1}{A}_{1}+{\beta }_{2}{A}_{2}+\mathrm{...}+\,{\beta }_{i}{A}_{i}+\varepsilon $$

where *Income* refers to the household income value of a ward in London, and *A*_*i*_ represents the percentage of an amenity number in a particular ward. To deal with the skewed distribution of household income, we apply logarithmic transformation to the values of household income in order to learn a more robust regression model.

Similarly, by only taking typeface as the explanatory variables, similary, the household income is modeled as:

2 $$Type\,face\,model(Model\,2):\,log(Income)={\beta }_{0}+{\beta }_{1}{T}_{1}+{\beta }_{2}{T}_{2}+\mathrm{...}+\,{\beta }_{i}{T}_{i}+\varepsilon $$

where *T*_*i*_ refers to the percentage of an typeface number in a particular ward.

Finally, by involving both typefaces and amenities into the household income model, we have:

3 $$Combined\,model(Model\,\mathrm{3)}\,:\,log(Income)={\beta }_{0}+\mathop{\sum }\limits_{n\mathrm{=1}}^{i}\,{\beta }_{i}{T}_{i}+\mathop{\sum }\limits_{n\mathrm{=1}}^{j}\,{\beta }_{j}{A}_{j}+\varepsilon $$

where *T*_*i*_ is the explanatory variables and *A*_*i*_ serves as the controlled variable.

**Figure 3:**
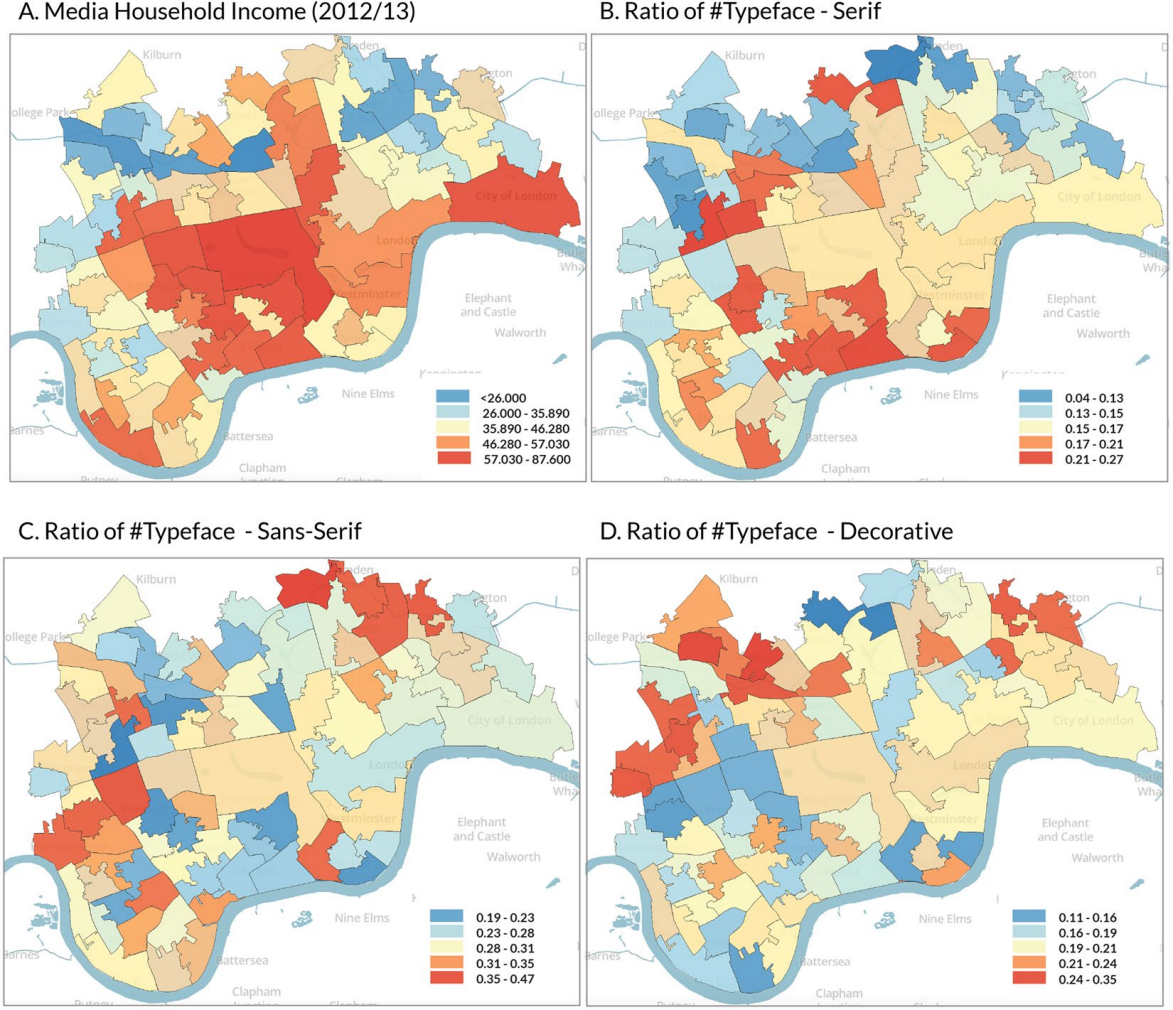
Spatial distribution of (**A**). Media household income; (**B**–**D**) the ratio of three typefaces, namely Serif, Sans Serif, and Decorative in each ward.

In order to measure the maximum degree of the relationship between typeface/amenity information and household incomes, we use the whole sample of typeface data (59,515) and amenity data (21,905). Fig. 3 depicts the spatial distribution of median household income and three typefaces. For typeface, the value is calculated by the ratio of the number of a typeface to the total amount of all typefaces of a ward. We can see that the distributions of the three typefaces present clear spatial patterns and are varying from each other.

Table 1 presents the detailed coefficients and *R*^2^ of the three model. Generally, When combining amenity types and typeface data, they explain household income better (*R*^2^ = 0.552) than using the typefaces (*R*^2^ = 0.410) or amenity types (*R*^2^ = 0.297) individually. The determination coefficients *R*^2^ generally indicate that variation of household income can be better explained by typeface information than using amenity information (0.410 V.s 0.297); and typeface can explain extra variation of household income in addition to amenity information (0.552V.s 0.297).

**Table 1:** Multivariate regressions between household income and amenities/typefaces.

Type	Variable	Model 1		Model 2		Model 3	
		Amenity Model		Typeface Model		Combined Model	
		Coeff.	Std. Coeff.	Coeff.	Std. Coeff.	Coeff.	Std. Coeff.
Amenity	Beauty Salon	10.481***	0.277***			5.019***	0.457***
	Café	10.518***	0.207***			4.723***	0.505***
	Clothing Store	11.854***	0.247***			5.471***	0.546***
	Electronics Store	10.985***	1.114***			6.167***	1.008***
	Finance	10.923***	0.25***			5.602***	0.456***
	Home Goods Store	10.766***	0.164***			4.941***	0.485***
	Night Club	9.366***	1.784***			4.287***	1.541**
	Pharmacy	10.366***	0.587***			4.635***	0.622***
	Restaurant	10.657***	0.09***			4.838***	0.467***
	Supermarket	10.247***	0.343***			5.046***	0.579***
Typeface	Blactletter			17.615***	4.403***	11.299***	4.094**
	Bold Slab Serif			5.541***	6.341	−2.636***	6.124
	Bold Sans Serif			7.532***	0.804***	2.98***	1.043**
	Bold Serif			9.423***	1.08***	3.249***	1.381*
	Slab Serif			11.102***	1.665***	6.902***	1.71***
	Casual			8.903***	1.323***	3.647***	1.359**
	Decorative			10.782***	0.342***	5.92***	0.646***
	Sans Serif			10.74***	0.281***	5.81***	0.534***
	Script			14.429***	1.097***	9.15***	1.28***
	Bold Script			5.417***	3.186	−1.932***	3.202
	Serif			11.283***	0.457***	6.339***	0.714***
*R*^2^		0.297***		0.410***		0.552***	

Model 1: taking amenity as the explanatory variable; Model 2: taking typeface as the explanatory variable; Model 3: taking both amenity and typface as the explanatory variable. Model 2 outperforms model according to *R*^2^. ***p < 0:001, **p < 0:01, * p < 0:05.

**Figure 4:**
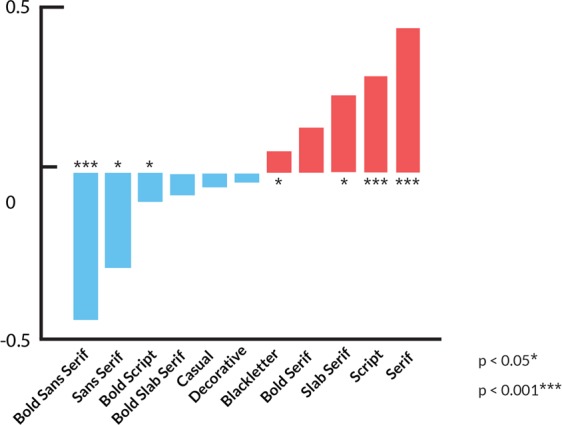
Spearman correlation coefficients between household income and typefaces.

Considering the potential multicollinearity among the amenity and typeface variable, as well as the skewed value distribution of amenity and typeface numbers, we didn’t look into the coefficients of the regression model since it is potentially biased when compare with each other. In order to identify the actual contributions of different typefaces to household income, we further employed Spearman’s ranking method to obtain the correlation coefficients between household income and each typeface category. A total size of 59,515 typefaces are involved in the correlation analysis. As shown in Fig. 4, Serif evidently has the highest correlation coefficient with household income (*ρ* = 0.44), followed by Script (*ρ* = 0.23). By contrast, Sans Serif has a negative correlation with household income (*ρ* = −0.26). This correlation represents that high-income people live in wards with a large number of Serif and Script typefaces. Thus, we presume that reassuring and readable impressions are popular in high-income areas. Notably, not all types of typefaces have significant coefficient with incomes. For example, Decorative, Casual, and Blackletter have very low correlation coefficients with income.

From these experiments, we can take typeface as an important aesthetic element to understand cities. Apart from its artistic influence on our urban city, it also correlates significantly with amenity types, household incomes and other socio-economic characteristics.

## Discussion and Conclusion

This research contributes to the following directions. First, we proposed a state-of-the-art framework to recognize typeface from Google Street View images. The framework also successfully maps the typeface in the Street View images to its corresponding amenity attributes. Second, we used the collected data to examine the relationship between typefaces and amenity types in a quantitative manner, which can only have an qualitative discussion before. For example, Decorative and Script are highly correlated with nightclubs, Sans Serif and Serif correlate with the finance industry. Our results provide some empirical evidence of how the usage of typeface is linked to the function of an amenity. Finally, we find that typefaces can contribute to the explanation of the local socio-economics, and different typefaces have different correlation coefficients to the economy. We mapped 11 typeface class correlation coefficients with household income.

Nevertheless, these results should be taken as preliminary, where we acknowledge two limitations - sample data uncertainty and the lack of multiple area analysis, which reduces the extent the research results can be generalized. Regarding the data uncertainty issue, owing to the fast development of machine learning methods, many studies employ the data yield from a machine learning model and attempt to explore the associations between predicted data and others. However, how to involve the uncertainty of samples to the statistical analysis is worth discussing. This work also faces the issue when employing the typeface data generated from machine learning models. This issue can be potentially approached through two ways: first, modeling and including errors as a kind of effect in a special statistical model; second, obtaining the results from different machine learning models and comparing the results.

Besides, in future work, we will apply our method to other cities worldwide to test the generalizability and transferability. We believe that different cities will have different typeface patterns, and the relationship between the typefaces and socio-economic indicators may vary. Exploring how different cities’ incomes are affected by typefaces is promising. Another interesting topic is to look further on whether typeface have an impact on other social indicators in our cities, such as sense of safe, human activities, demographics, or even political preferences. For example, election posters can influence the election results^23^. We believe that political preferences could be revealed via typefaces. Therefore, a strong potential arises in using typeface as a key to unlock many of the still unknown demographic indicators by combining solid spatial statistical and geographic modeling methods^24,25^. Moreover, typeface usage patterns could also be beneficial to the linguistic landscape field, such as Cook’s the language of street study^26^. Last but not least, we hope our method can help other urban planners, designers, and engineers to reveal the city with their interest.

### Methods for typeface dataset collection and mapping of amenity attributes

**Figure 5:**
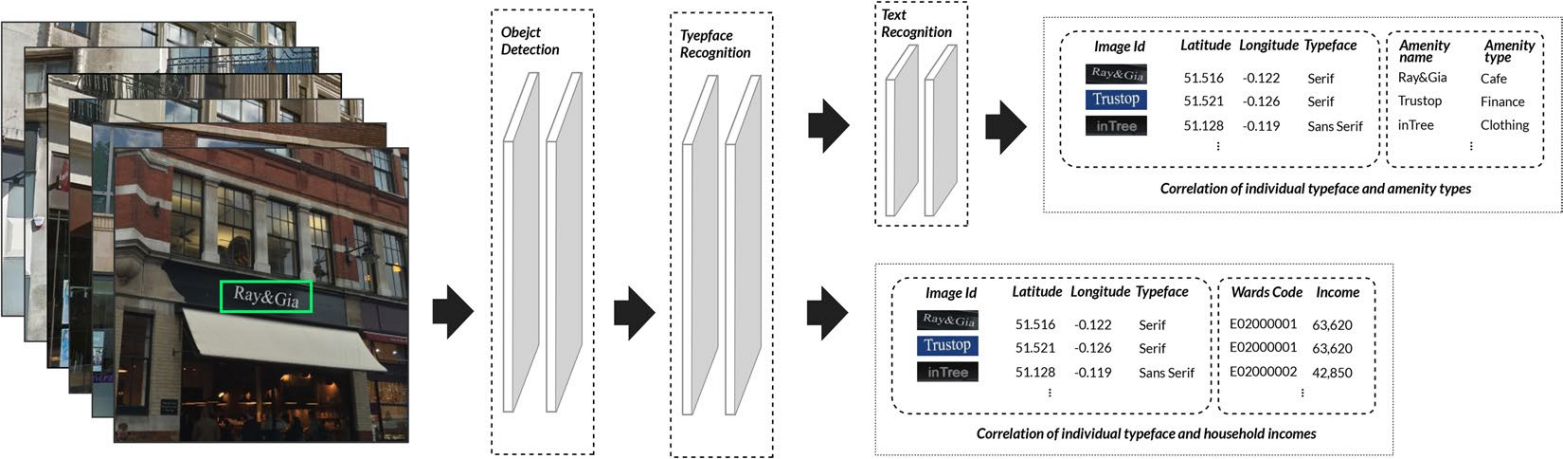
Dataset collection work flow (Due to copyright, our example of Street View photo was taken by our-self instead of actual Google Street View).

Fig. 5 presents the working pipeline in this research. To generate ground truth typeface data, we use a learning-based pipeline to build our study, which requires three steps to complete our experiment:

- *Text detection from the Street View image*. Extract localization of the text in the Google Street View image, and retain the geographic information of these Street View images, such as latitude and longitude, and so on.
- *Typefaces recognition*. Identify typefaces from the text images that just extracted from Google Street View images, and retain geographic information of the text images.
- *Match typeface with amenity name*. We recognize the semantic text using the text images from the last step and continue to preserve the typeface, geographic information, and so on. Then, we match the name of the amenity with the text identified from the text image to obtain the amenity information corresponding to the text image.

These three steps use three different models to synthesize the pipeline as shown in Fig. 5, namely, object detection, typeface recognition, and text recognition models. The following three sections describe how to use these models in each step, respectively. All of our visual analytic datasets are based on Google Street View images.

The Street View Images can be downloaded through the Google Street View Application Programming Interface (API). The API is an HTTP URL that allows users to modify the attributes by Latitude and Longitude, heading (compass of the camera, range from 0 to 360), fov (determines the horizontal field of view of the image), and pitch (specifies the up or down angle of the camera). According to Li’s study with regard to collecting Google Street View images^27^. We set the position parameters of our API according to the path of the street, because most signage and posters are placed on buildings and because the Street View Image resolution is limited (640 × 640). Hence, in our test, when setting the pitch to 11 and fov to 45, the signage is likely captured in the image. In the meantime, while most studies set six headings to obtain a 360° panorama Street View Image^6,27^ on one site, we divide 360° into eight headings so that each captured Street View image will have sufficient pixels for further analysis. In addition, we set Global Positioning System (GPS) coordinates every 15 m to capture valid signages and posters on the street. A distance longer than this set up would miss capturing shops in high-density store areas, and a shorter distance would increase the chances of repeating signages. In total, we set 97,154 sites and collected 748,471 street view images (some places have no valid street images) in central London as of November 2016.

### Text detection from street view images

To collect the text localization data of street view images from more than three quarter million images, we use an Efficient and Accurate Scene Text detector (EAST)^28^; it can extract the localization of the text area in the street view images. Then, we spend about 42 hours to run all 748,471 images on an NVIDIA GeForce GTX 1080. While collecting street view images from London, except amenity’s appearance, we also collected several residential house images, and the balcony in these residential images are often considered as text by recognition network. This situation also happens when we test on other networks such as Faster R-CNN network^29^. Therefore, we trained a neural network to filter residential houses, which include balcony images. We fine-tune a ResNet-101 model and obtain an accuracy of 93% with 84% recall. Under such conditions, we finally retrieved 59,515 text images through 748,471 Google Street View images from London. Although some biases remain in this recognition, the accuracy is acceptable for further analysis.

### Typeface recognition

Supervised learning such as the deep learning requires a large amount of ground truth data. However, collecting typeface data is time consuming and expensive. Even accurately labeling typeface data would require professional knowledge. Therefore, synthesizing text images would be our best option to obtain adequately detailed annotation of the typeface dataset.

**Figure 6:**
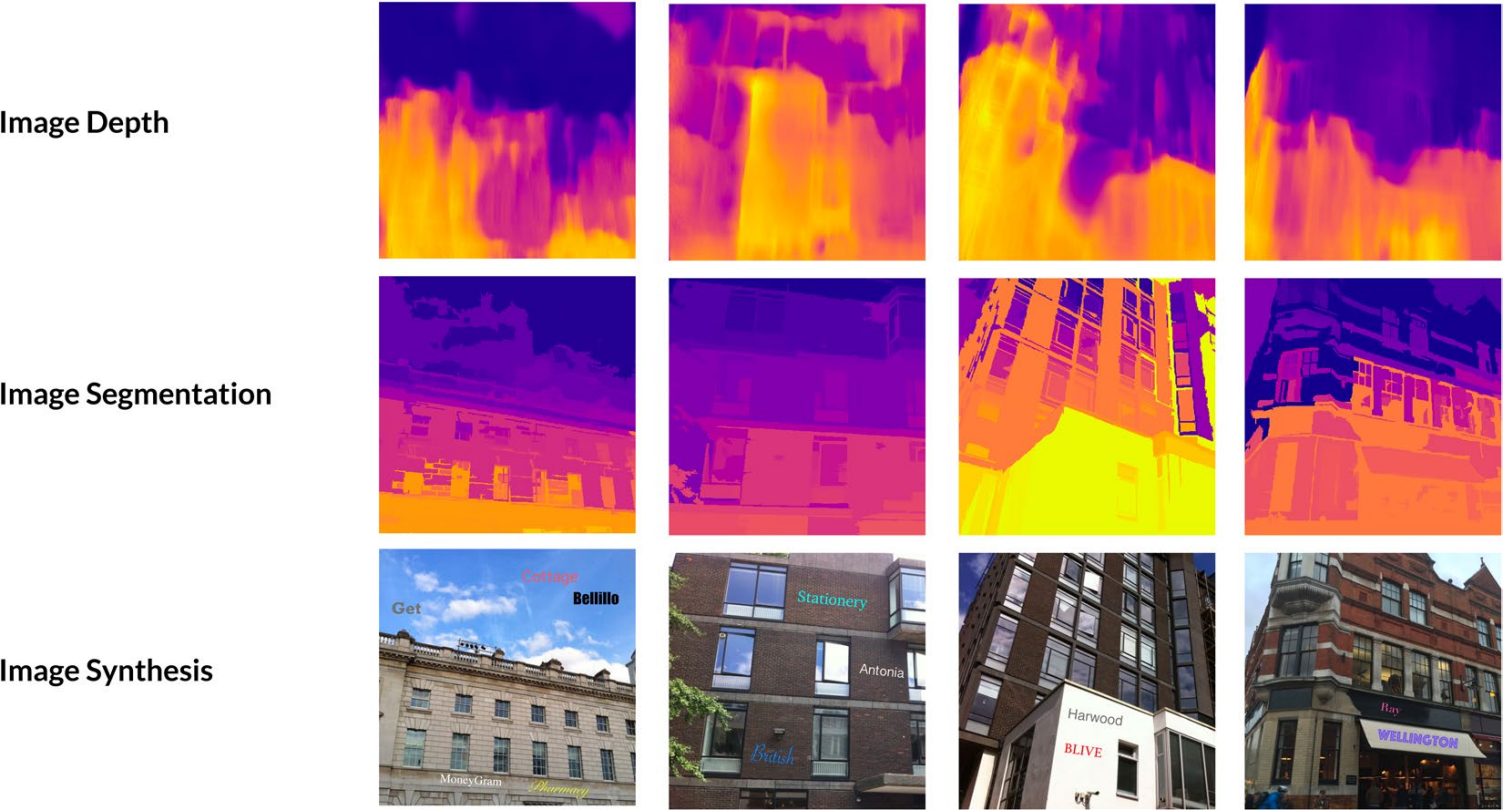
Building typeface training set: synthesizing typefaces on natural images based on segmentation and depth information.

We followed Gupta’s^30^ synthetic approach to generate text on the buildings’ outdoor images. The essential workflow is to determine images without text in the image and then identify an appropriate space to place the text. Technically, synthesizing text on the image is based on the result of image segmentation to determine a region with sufficient continuities to place text. Moreover, the depth data of the image can change text distortion and transform it according to the normal surface of the region. We use pre-generated data by Gupta, which rely on Arbelaez’s segmentation data^31^ and Liu’s depth data^32^. Some examples of synthesized images can be found in  Fig. 6. Based on these data, we could finally add typeface as one additional attribute to Gupta’s implementation^30^; the typeface class label is selected from our 11 typeface classes. Therefore, when we synthesize an image, we can obtain the text typeface and localization in an image. We then use this method to generate 91,398 text images as our ground truth images. Through the generating process, we use amenity names downloaded from the Google Shops API as the text letters to synthesize on the images, and each amenity name is considered as a text area. Following this method, we generate text on the possible segmentation area in the image.

**Figure 7:**
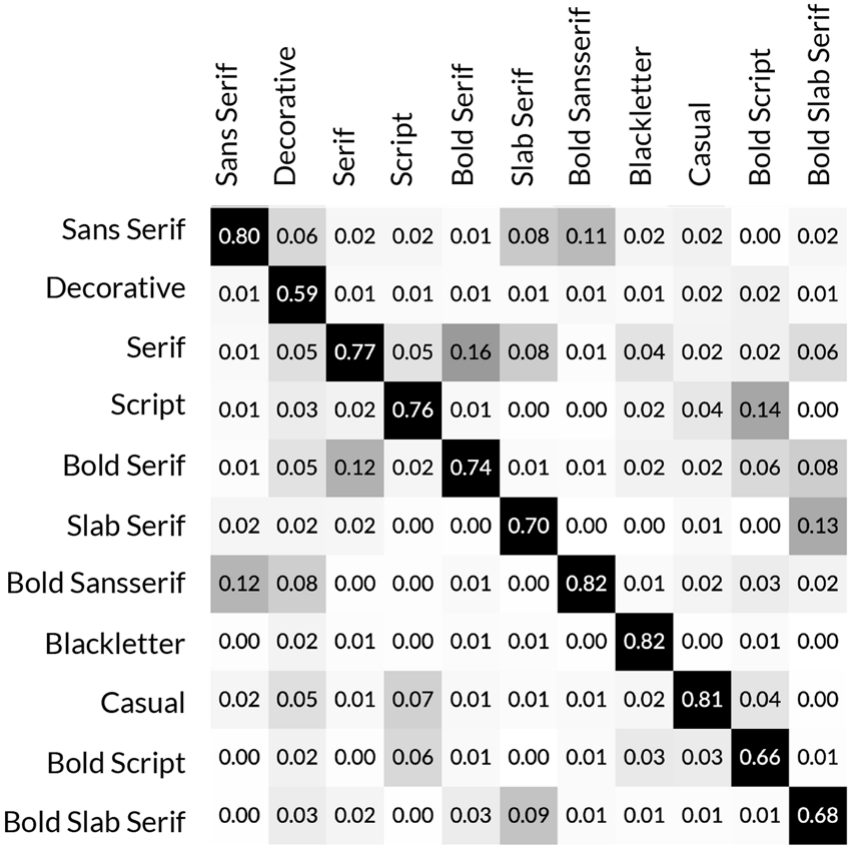
Confusion Matrix of 11 typefaces recognition result.

Once we successfully synthesize the data, we fine-tune a ResNet-18 network^33^ that pretrained on ImageNet dataset^34^. The model achieved higher accuracy for the typeface recognition and saved a large amount of time rather than training the network from scratch. The dataset is splited into training set (80%) and testing set (20%). In the training process, we set the learning rate to 0.001, momentum to 0.9, and weight decay to 0.0001. The optimization took 50 epochs and obtained an accuracy of 76% mean accuracy for the 11 classes on the test dataset. Fig. 7 presents the confusion matrix of the classification model.

### Match typeface with amenity attributes

The amenity type data, or similarly known as “Point of Interest” data, has been used widely to help evaluate the development and improvement of urban built environment^35^. However, given that collecting the amenity typefaces is extremely difficult, few studies have been conducted to understand a city through the perspective of amenity typefaces. With the text image data we obtained from the previous steps, we build a mapping system to match the text image to its corresponding amenity properties. As two preparatory steps, the details are shown below:

- *Text recognition* We use the Google Street View images as input images to train a Convolutional Recurrent Neural Network (CRNN)^36^ to recognize text characters; then, we can create a text image dataset with corresponding geo-locations.
- *Amenity name collection* We use the Google Places API to collect the ten most common amenity categories in the city. Totally, there are 21,905 shops being obtained, with their detailed information including locations and categories. According to^35^, the average amenity number in the city is around 26,800. In addition, considering the area we focused is only the city center of London, we believe that this amount of amenities cover more than 80% of the amenities in the city.

Finally, we build a mapping system to link the amenities’ names we collected from the Google Places API and the text images. The system works as follows: choosing a text image with a geographic location and recognized letters; then, use the location of this text image as the center of a 50-m circle. Then, we attempt to use the recognized word to match every amenity’s name. For example, we have a text image in location A, and we recognized it by using the CRNN network^36^ to obtain the letters, such as “Burberr” From Google Places API, we know that an amenity named Burberry exists within 50 m of this text image. The letters “Burberr” have a high possibility of being Burberry. Thus, we match the text image “Burberr” with the amenity “Burberry”. Therefore, we can also obtain all related amenity information of this text image “Burberr” such as the category of the amenity and the GPS coordinates. Through this method, we successfully retrieved 5,238 text images with corresponding amenity attributes.
