# Author Correction: Typeface Reveals Spatial Economical Patterns

**DOI:** 10.1038/s41598-019-55789-1

**Published:** 2019-12-24

**Authors:** Ruixian Ma, Wei Wang, Fan Zhang, Kyuha Shim, Carlo Ratti

**Affiliations:** 1 Senseable City Laboratory, Massachusetts Institute of Technology, Cambridge, MA 02139 USA; 2 Alibaba Group, Hangzhou, 310000 China; 3 School of Design, Carnegie Mellon University, Pittsburgh, PA 15213 USA

Correction to: *Scientific Reports* 10.1038/s41598-019-52423-y, published online 04 November 2019

The original version of this Article contained an error in Affiliation 3, which was incorrectly given as ‘School of Design, Carnegie Mellon University, Pennsylvania, PA, 15213, USA.’ The correct affiliation is listed below:

School of Design, Carnegie Mellon University, Pittsburgh, PA, 15213, USA.

This error has now been corrected in the HTML and PDF versions of the Article.

